# Datasets for characterizing extreme events relevant to hydrologic design over the conterminous United States

**DOI:** 10.1038/s41597-022-01221-9

**Published:** 2022-04-05

**Authors:** Ning Sun, Hongxiang Yan, Mark S. Wigmosta, Andre M. Coleman, L. Ruby Leung, Zhangshuan Hou

**Affiliations:** 1 Energy and Environment Directorate, Pacific Northwest National Laboratory, Richland, Washington United States; 2 Department of Civil and Environmental Engineering, University of Washington, Seattle, Washington United States; 3 Earth and Biological Sciences Directorate, Pacific Northwest National Laboratory, Richland, Washington United States

**Keywords:** Hydrology, Hydrology

## Abstract

Despite the close linkage between extreme floods and snowmelt, particularly through rain-on-snow (ROS), hydrologic infrastructure is mostly designed based on standard precipitation Intensity-Duration-Frequency curves (PREC-IDF) that neglect snow processes in runoff generation. For snow-dominated regions, such simplification could result in substantial errors in estimating extreme events and infrastructure design risk. To address this long-standing problem, we applied the Next Generation IDF (NG-IDF) technique to estimate design basis extreme events for different durations and return periods in the conterminous United States (CONUS) to distinctly represent the contribution of rain, snowmelt, and ROS events to the amount of water reaching the land surface. A suite of datasets were developed to characterize the magnitude, trend, seasonality, and dominant mechanism of extreme events for over 200,000 locations. Infrastructure design risk associated with the use of PREC-IDF was estimated. Accuracy of the model simulations used in the analyses was confirmed by long-term snow data at over 200 Snowpack Telemetry stations. The presented spatially continuous datasets are readily usable and instrumental for supporting site-specific infrastructure design.

## Abstract (summary)

**Table:** 

Measurement(s)	gridded precipitation
Technology Type(s)	weather station
Sample Characteristic - Environment	flood • snowmelt • hydrological process
Sample Characteristic - Location	contiguous United States of America

## Background & Summary

Although it is well understood in the scientific community that extreme hydrometeorological events in cold climates are often related to snow processes, i.e., snowmelt and rain-on-snow (ROS)^1–6^, they have largely been ignored or under-represented in hydrologic design that relies largely on traditional precipitation-based intensity-duration-frequency curves (PREC-IDF) to estimate design basis extreme events (e.g., 100-year 24-hour event). PREC-IDF such as the National Oceanic and Atmospheric Administration (NOAA) Atlas 14^7^ assumes that precipitation is in the form of rainfall that is immediately available for rainfall-runoff processes. This assumption has obvious shortcomings, especially in snow-dominated regions where winter precipitation is primarily snowfall. At locations where runoff is released slowly from accumulated snowpack, PREC-IDF can lead to infrastructure overdesign and incur unnecessary costs. Conversely, underdesign will occur where the snow-driven runoff rate is higher than the rate of precipitation. This was confirmed by previous research^8,9^, which demonstrated that PREC-IDF underestimates the 100-year, 24-hour extreme events in 45% of the Snow Telemetry (SNOTEL) stations examined, and the resulting peak design flood could be underestimated by up to 324%.

The Oroville Dam failure in February 2017 that required $1.5 billion to repair is a notable example of costly infrastructure damages resulting from floods driven by ROS events^10^. The exclusion of snow processes in the PREC-IDF technique is likely to cause greater errors in estimating extremes in a warming climate and present higher infrastructure design risk. For example, increased frequency and intensity of atmospheric rivers in the future are anticipated to cause more extreme orographic precipitation and subsequently increase flood risk along the U.S. West Coast^11–14^.

Given the limitations in PREC-IDF and implications for design risk, the Next-Generation IDF (NG-IDF) technique^8^ was developed to estimate extreme events based on the amount of water reaching the land surface (*W*) during rain, snowmelt, and ROS events. By including snow processes, NG-IDF provides a systematic and consistent technique for all environments from rain-dominated, transitional, to snow-dominated locations. Despite the marked advantages of NG-IDF over PREC-IDF, its wide adoption by engineers and planners is hindered by rather limited snow observations for estimating *W*, especially relative to widely available precipitation products^15–17^. Using physics-based models to produce reasonable simulations of *W*, while feasible, is rather challenging, given the significant requirements of expert knowledge in model calibration and considerable cost of computational resources.

To support the broad adoption of the NG-IDF approach, we developed a suite of datasets that characterize extreme events relevant to hydrologic design over 1951‒2013 at a 1/16th-degree (~6 km) resolution across the conterminous United States (CONUS). For over 200,000 locations in the CONUS, the datasets provide: the magnitude and dominant driving mechanism (i.e., rain, melt, and ROS) of extreme events for different durations (24‒72 hours) and return periods (2‒500 years) derived from the NG-IDF curves; the magnitude of design extreme events associated with different hydrometeorological drivers; trend and seasonality of annual maximum *W* events (AMW) over 1951‒2013; and infrastructure design risk associated with the PREC-IDF technique. These datasets were developed based on sub-daily simulations of *W* by a well-validated physics-based hydrological model (Distributed Hydrology Soil Vegetation Model, DHSVM^18^). To examine the accuracy of the simulations, the simulated snow water equivalent (SWE) was evaluated against long-term observations of SWE from 246 SNOTEL stations distributed across the Western U.S. These datasets intend to offer spatially distributed, quantitative measures of extreme events that are readily usable by the science and engineering communities to understand the climatology and driving mechanism(s) of extreme events, identify potential infrastructure design risk associated with the standard PREC-IDF method, and improve estimation of extremes.

## Methods

### Overview of NG-IDF datasets development

**Fig. 1:**
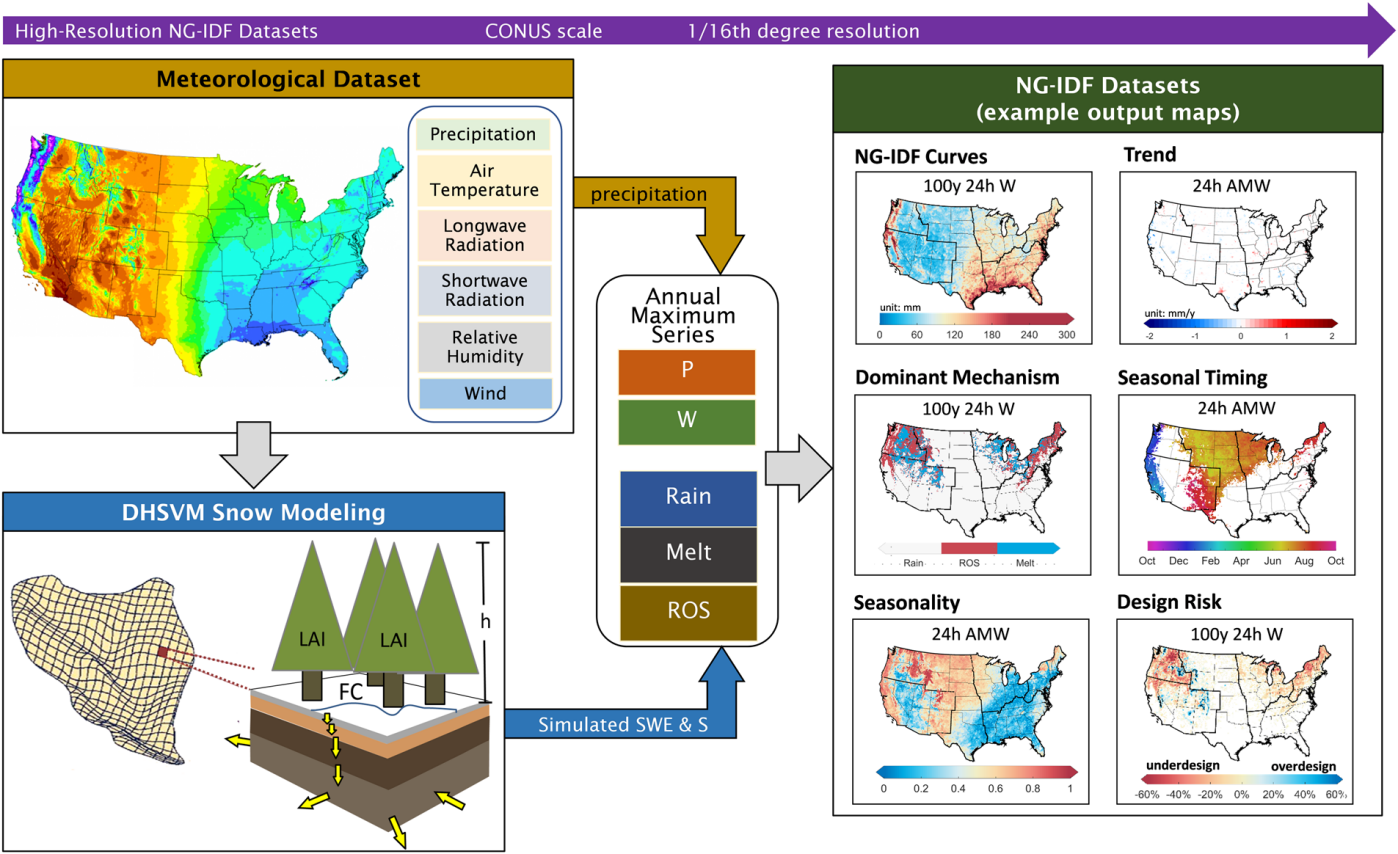
Schematic view of the NG-IDF datasets.

The method to develop the NG-IDF datasets is illustrated in Fig. 1. In contrast to PREC-IDF that estimates extreme events based on the total amount of precipitation (implicitly assumed to be in the form of rain), NG-IDF curves are developed based on the amount of water available for runoff (*W*) for the bare ground condition with no canopy cover, which can be represented mathematically by:

1 $$W=P-\Delta SWE+S$$

where *P* is precipitation, Δ*SWE* is the change in ground snowpack water content, and *S* indicates condensation (positive) or evaporation/sublimation (negative) of snowpack. In this development, as described in more detail in the following sections, *P* was taken from the gridded, gauge-based meteorological dataset, Δ*SWE* and *S* were simulated by the DHSVM snow model. The resulting *W* data is a 3-hourly time series for 1950–2013 at 207,173 grid cells covering the land surface of the CONUS at a 1/16th-degree resolution.

As observations suggested that snowfall events largely last up to 72 hours^19^, NG-IDF curves were developed for *W* events with 24-, 48- and 72-hour durations, respectively. We first developed series of annual maximum *W* (AMW) for years 1951–2013 based on subdaily estimations of *W* aggregated over different durations. For example, AMW with a 72-hour duration is the maximum of the 72-hour moving sum of *W* over a given year. Given variations in precipitation seasonality across the CONUS, we defined AMW events for both water year (October 1st through September 30th) and calendar year (January 1st through December 31st). AMW based on calendar year is more appropriate for locations with extreme precipitation occurring during the summer season such as the Southwest U.S., and AMW based on water year is better for locations with extreme winter precipitation such as the Western U.S. Given the focus on snow-driven extreme events, we used AMW defined by water years for IDF curves.

We applied the nonparametric Mann-Kendall test^20,21^ on the AMW series. Where trends were significant at the 5% confidence level, we detrended the time series using Sen’s slope^22^ while maintaining the long-term average of the time series. As suggested by NOAA Atlas 14, the generalized extreme value (GEV) distribution was fitted to AMW across all locations using the L-moments statistics^7,8^. Here we used the same GEV distribution for analysis of extreme events with different durations across the CONUS so that direct comparisons can be made in frequency estimates across durations or between locations. To quantify the implications of using PREC-IDF for infrastructure design risk, we also developed PREC-IDF curves following the same approach based on annual maximum precipitation (*P*). The design risk was quantified by comparing the NG-IDF and PREC-IDF values of extreme events (e.g., 100-year 24-hour event) over the CONUS.

A Monte Carlo (MC) simulation procedure^23^ and NOAA Atlas 14^7^ was used to consider sample data uncertainty in frequency analysis. For each location, we generated 1,000 MC synthetic annual maximum series. We then fitted the GEV distribution to each MC series using the L-moments statistics and estimated the associated NG-IDF values. The uncertainties in NG-IDF curves for each location were quantified using the 5% and 95% quantiles (i.e., the 90% confidence interval) of ensemble members.

### Gauge-based gridded meteorological data

For simulations of snow processes, the model requires subdaily input of *P*, air temperature, relative humidity, downward shortwave and longwave radiation, and wind speed. The meteorological input used here was derived from disaggregating the gridded (~6 km), daily land surface meteorological dataset^24^ over the CONUS using the Mountain Microclimate Simulation Model (MTCLIM) algorithm^25^. Distinct from reanalysis products, this dataset is one of the few gauge-based gridded datasets with long-term continuous records. It includes daily records of *P*, maximum and minimum air temperature, and wind speed spanning the period 1950–2013. The disaggregation algorithms^25^ estimate subdaily air temperatures with a 3rd-order polynomial fit based on daily temperatures range. Radiation and relative humidity are estimated based on daily temperature range, *P*, and solar geometry^25^. *P* is assumed to occur at a uniform rate throughout the day.

### Observational snow data

Daily SWE measurements were retrieved from SNOTEL stations for snow model parameterization and evaluation. Among the 785 SNOTEL stations, we selected 246 stations that shared the longest common period (2007‒2013) of bias-corrected and quality-controlled (BCQC) daily SWE records. Briefly, standard quality control procedures^8,26^ were applied to remove stations with missing data, outliers, and problematic SWE values (e.g., peak SWE > accumulated winter precipitation). The BCQC procedures and the resulting BCQC datasets^27^ are available to the public at https://www.pnnl.gov/data-products.

### CONUS-scale snow modeling and parameter development

The physics-based, snow submodel of DHSVM^18^ was implemented at the point scale to simulate snowpack dynamics under a bare ground and flat terrain condition. The model was run at the 3-hourly time step from 1950–2013 for 207,173 point locations at a 1/16th-degree grid spacing that coincides with the center of the meteorological grids. Model output includes 3-hourly times series of SWE and *S*, which are used together with observed *P* for calculating *W* in Eq. 1.

DHSVM simulates ground snowpack accumulation and melt using a two-layer mass and energy balance ground snowpack module. The mass balance components consist of *P*, *S*, changes in *SWE*, and melt from the snowpack. The partition of *P* into rain and snow is based on air temperature thresholds:

2 $$\left\{\begin{array}{cc}R=P & {T}_{a}\ge {T}_{R}\\ R=P({T}_{R}-{T}_{a})/({T}_{R}-{T}_{S}) & {T}_{S} < {T}_{a} < {T}_{R}\\ R=0 & {T}_{a}\le {T}_{S}\end{array}\right.$$

where *T*_*a*_ is air temperature, *T*_*S*_ and *T*_*R*_ is the temperature threshold for *P* to be completely snowfall and rain, respectively. If *T*_*a*_ ≥ *T*_*S*_, 100% of the *P* is rain (*R*); if *T*_*a*_ < *T*_*R*_, 100% of the *P* is snow; if *T*_*a*_ falls between the two thresholds, rain and snow are proportionally allocated to represent mixed rain and snow events. Energy balance at the snow surface is driven by net radiation, sensible and latent heat, and advected heat by rain. Energy and mass exchange between the thin surface layer and deep snowpack layer occurs via the exchange of meltwater. When liquid water in the deep snowpack exceeds its holding capacity, excess water is released to the underlying soil column. Detailed descriptions of the DHSVM snow model physics and governing algorithms can be found in a large body of literature^18,28–30^.

**Fig. 2:**
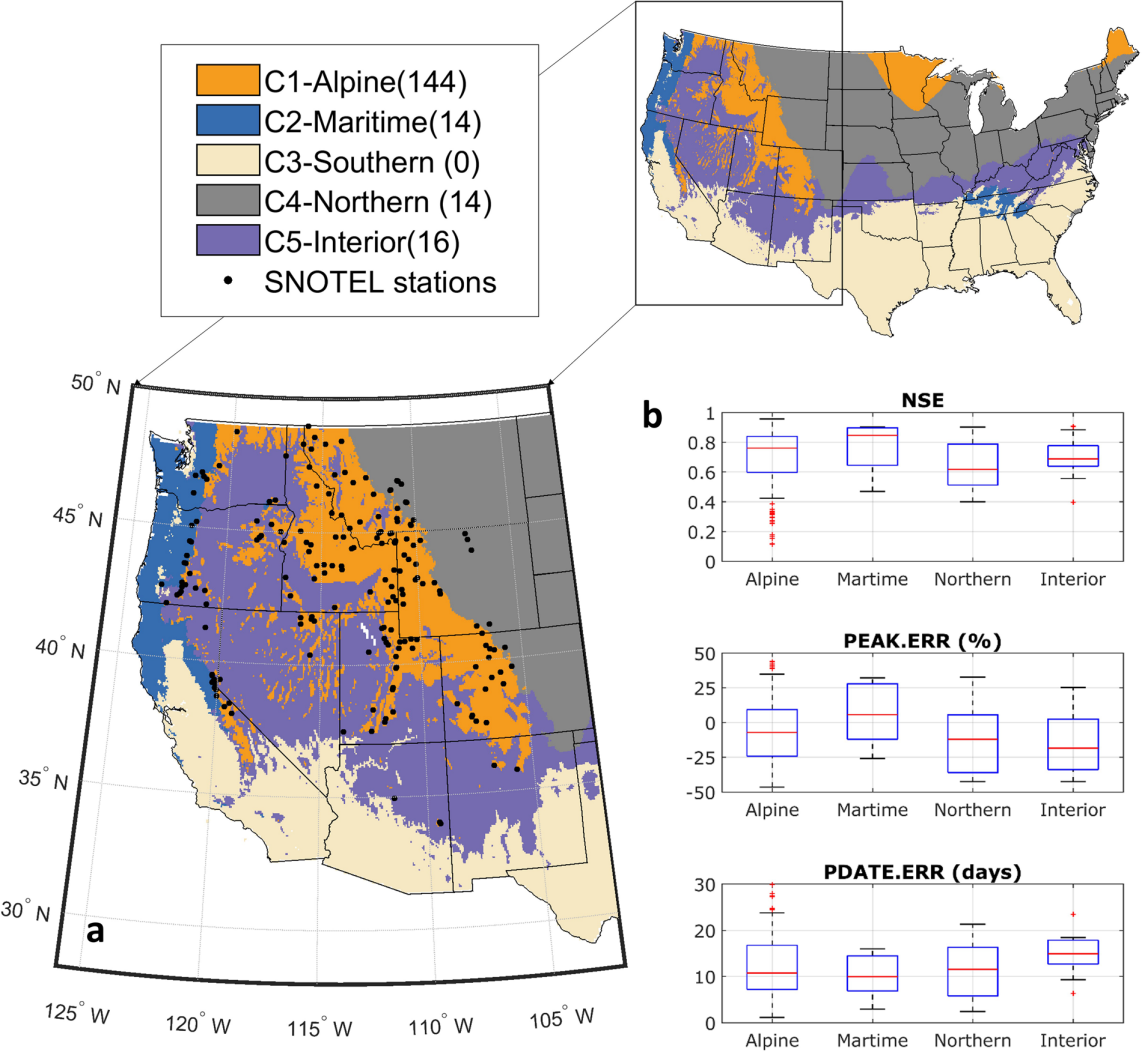
Evaluation of cluster snow parameters in snow modeling. (**a**) Five clusters grouped by the mean winter precipitation, air temperature, and wind speed at the 1/16° resolution in the CONUS. Numbers in the parenthesis following each cluster name indicate the number of SNOTEL stations (black dots) within the cluster used for parameter development; (**b**) boxplots showing the model performance against SWE observations from SNOTEL stations, which was measured by NSE, PEAK.ERR and PDATE.ERR and grouped by clusters.

Prior calibration of snow models is performed typically at relatively local scales; thus, there are no calibrated, spatial snow parameter sets that can be readily applied for the CONUS-domain snow modeling. In support of this work as well as future large-domain snow modeling, here we developed spatially distributed snow parameters for the CONUS domain. Based on previous research^29^ that documented the robustness of regionally coherent snow parameters in modeling snowpack dynamics, we developed snow parameters for five spatial clusters covering the CONUS (Fig. 2). Given strong correlations between winter climate and key aspects of snowpack dynamics^31–33^, we determined the clusters using the k-means clustering machine learning technique^34^ based on the grid-level climatological mean of *P*, maximum and minimum air temperature, and wind speed from November through March during 1950–2013. Different numbers of clusters were tested and we selected the optimal five clusters based on the inertia elbow method and our previous work^29^.

Parameter development focused on four snow parameters that were identified by previous work^29^ to be crucial for capturing daily SWE dynamics^29^: (1) *T*_*S*_ (defined in Eq. 2), (2) fresh snow albedo (*a*_*max*_), (3) albedo decay coefficient during snow accumulation (*λ*_*A*_) and (4) snowmelt (*λ*_*M*_). The last three parameters are applied in the snow albedo decay curve of Laramie & Schaake^35^ for estimating snow surface albedo evolution, given by:

3 $$\begin{array}{lll}{\alpha }_{A} & = & {\alpha }_{max}\cdot {\lambda }_{A}^{{d}^{{\rm{0}}{\rm{.58}}}}\\ {\alpha }_{M} & = & {\alpha }_{max}\cdot {\lambda }_{M}^{{d}^{{\rm{0}}{\rm{.46}}}}\end{array}$$

where *α*_*A*_ and *α*_*M*_ are the snow surface albedo during the accumulation and melting seasons, respectively, and *d* is the number of days since the last snowfall. For other model parameters, we used the default values. The cluster-based snow parameters (Table 1) were developed as follows: (1) we produced prior ensemble parameters, consisting of 10,000 sets of the four snow parameters drawn uniformly from their physically plausible ranges, using the Latin Hypercube Sampling algorithm; (2) snow simulations were conducted at each SNOTEL location for every prior parameter set; (3) the posterior ensemble parameters were resampled from the prior ensemble if they met the threshold values of objective functions with observations. Here, we used three metrics (Eqs. 4‒6): Nash-Sutcliffe Efficiency (NSE) of daily SWE ≥ 0.6, the bias in the mean annual peak SWE (PEAK.ERR) within ± 25%, and the bias in the timing of peak SWE (PDATE.ERR) ≤ 14 days.

4 $$NSE=1-\frac{{\sum }_{i=1}^{t}{({Y}_{i}-{O}_{i})}^{2}}{{\sum }_{i=1}^{t}{({O}_{i}-\bar{O})}^{2}}$$

where *O*_*i*_ and *Y*_*i*_ are the observed and predicted daily SWE at day *i*, respectively; *t* is the total number of days for which model simulations were performed; $\bar{O}$ is the observed mean daily SWE over the simulation period.

5 $${PEAK.ERR}={\sum }_{k=1}^{ny}\frac{({Y}_{k}^{P}-{O}_{k}^{P})}{{Y}_{k}^{P}}/ny$$

where ${O}_{k}^{P}$ and ${Y}_{k}^{P}$ are the observed and predicted peak SWE for the *k*^th^ water year, respectively; *ny* is the total number of years of simulation.

6 $${PDATE.ERR}={\sum }_{k=1}^{ny}\left({Y}_{k}^{D}-{O}_{k}^{D}\right)/ny$$

where ${Y}_{k}^{D}$ and ${O}_{k}^{D}$ are Julian dates of observed and predicted peak SWE for the *k*^th^ water year, respectively. After step (3), 58 stations with no qualified posterior parameter values were removed from subsequent analyses. Most of these stations are located in high-latitude areas of Montana and Wyoming, where model skill is challenged by the lack of representation of wind effects on snow redistribution, or the maritime Pacific Northwest where snowmelt tends to be sensitive to errors in modeled energy balance and precipitation partitioning when temperature is near freezing^29^; (4) for each cluster, final parameter values were calculated as the ensemble mean over the posterior parameter space of all stations within the cluster. For the Southern cluster (C3), no SNOTEL observations are available for parameter development. Because snowfall is very limited for most of the Southern cluster where snow parameters have negligible effects on extreme events, we used the parameter values of the maritime cluster for the Southern cluster given their commonality of warm winter. The cluster parameter values are presented in Table 1.

**Table 1:** Cluster snow parameters developed for the CONUS.

Cluster	Name	# SNOTEL	*α*_*max*_ [0.8, 0.9]*	*λ*_*A*_ [0.87, 0.99]*	*λ*_*M*_ [0.82, 0.99]*	*T*_*S*_ (°C) [0, 6.5]*
C1	Alpine	144	0.85	0.93	0.90	3.3
C2	Maritime	14	0.84	0.93	0.89	2.4
C3	Southern	0	0.84	0.93	0.89	2.4
C4	Northern	14	0.85	0.98	0.87	1.3
C5	Interior	16	0.84	0.93	0.84	2.0

Note: *prior range of snow parameters

### Driving mechanism of extreme events

For each location, the driving mechanism was determined for extreme *W* events with different durations and return periods. Table 2 presents the classification approach used for determining the mechanism based on subdaily information of *P* and *SWE*, which include:

1. Rainfall only (*R*): precipitation on snow-free ground;
2. Snowmelt only (*M*): decreasing *SWE* with no concurrent precipitation;
3. Rain-on-snow (*ROS*): decreasing *SWE* with concurrent precipitation. Given the interest in flood potential, a ROS event is further refined as one with at least 10 mm rainfall per day falling on a snowpack with at least 10 mm SWE over the selected duration, and the sum of rain and snowmelt contains at least 20% of snowmelt^36–38^.

**Table 2:** Classification of the driving mechanism of *W* extremes.

Driving mechanism	Classification criteria
Rainfall: *R*	if *SWE* = 0: *R* = *P*
Snowmelt: *M*	if *(∆SWE* < *0)* and *(P* = *0)*: *M* = *−∆SWE* + *S*
Rain-on-snow: *ROS*	if *(∆SWE* < *0)* and *(P* > *0)*: *ROS* = *P − ∆SWE* + *S*

Annual maximum series resulting distinctively from each driving mechanism were determined, based on which IDF curves were developed following the same approach as described for NG-IDF. The mechanism producing the largest IDF value was identified as the dominant mechanism of extreme events.

### Seasonality of extreme events

The seasonality of AMW at the 24-, 48- and 72-hour durations was represented by the seasonality index (*SI*) and the mean date (*MD*) relative to October 1st due to the use of water year. They were calculated using the circular statistics^1,39,40^, given by:

7 $$SI=\sqrt{{\bar{x}}^{2}+{\bar{y}}^{2}}$$

8 $$MD{=\tan }^{-1}(\bar{y}/\bar{x})\cdot 365/2\pi $$

where

$${\theta }_{i}=D\cdot 2{\rm{\pi }}/365$$

$$\bar{x}={\sum }_{i=1}^{n}\cos ({\theta }_{i})/n$$

$$\bar{y}=\mathop{\sum }\limits_{i=1}^{n}{\rm{\sin }}({\theta }_{i})/n$$

For a given water year denoted by *i*, D is the day of the AMF occurrence relative to October 1st (i.e., D = 1 if the event occurred on October 1st); *n* is the total number of water years used in the analysis. SI, ranging from 0 to 1, measures the temporal variability of the occurrence of events. A smaller SI suggests weaker seasonality, and the associated MD is therefore less reflective of the actual timing of the extreme events.

## Data Records

The NG-IDF datasets^41^ are available to the public through an unrestricted repository at 10.5281/zenodo.5827028 in comma-separated value (.csv) format. Table 3 provides a summary of the folder structures, description of data files, output variables in each file and the format.

**Table 3:** Description of the NG-IDF datasets.

Main Folder	Naming Convention	Data File Description*
	list.csv Description of 207,173 locations	**Data Dimension**: 207,173 (R) × 3 (C) **C1**: Latitude; **C2**: Longitude **C3**: Cluster ID (ranging from 1‒5) as defined in Table 1
AMF_WY/	Annual maximum series with durations of 24 h, 48 h, and 72 h, driven by different hydrometeorological mechanisms over water years 1951‒2013 (10/1/1950-09/30/2013). The mechanisms include W, P, R, ROS, and M as defined in Table 2.	
	[duration]/ [mechanism].csv e.g., 24 h/W.csv	**Data Dimension**: 207,173 (R) × 65 (C) **C1**: Latitude; C2: Longitude **C3‒C65 [unit: mm]**: Maximum value for each year from 1951–2013
AMF_CY/	Annual maximum series with durations of 24 h, 48 h, and 72 h, driven by different mechanisms (W, P, R, ROS, and M) over calendar years 1950‒2012.	
	[duration]/ [mechanism].csv e.g., 24 h/W.csv	**Data Dimension**: 207,173 (R) × 65 (C) **C1**: Latitude; **C2**: Longitude **C3‒C65 [unit: mm]**: Maximum value for each year from 1950–2012
IDF/	Discrete IDF values, i.e., the magnitude of extreme events with durations of 24 h, 48 h, and 72 h, driven by different hydrometeorological mechanisms (W, P, R, ROS, and M).	
	[duration]/ [mechanism].csv e.g., 24 h/W.csv	**Data Dimension**: 207,173 (R) × 9 (C) **C1**: Latitude; C2: Longitude **C3‒C9 [unit: mm]**: IDF values for the return period of 2, 5, 10, 25, 50, 100, and 500 years. NaN indicates no runoff caused by a given mechanism, such as ROS.
IDF_90CI/	90% C.I. for IDF values in the IDF/ folder described above	
	[duration]/ [mechanism]_H.csv e.g., 24 h/W_H.csv	**Data Dimension**: 207,173 (R) × 9 (C) **C1**: Latitude; **C2**: Longitude **C3‒C9 [unit: mm]**: 95% quantile for IDF values with the return period of 2, 5, 10, 25, 50, 100, and 500 years. NaN indicates no runoff event caused by a given mechanism.
	[duration]/ [mechanism]_L.csv e.g., 24 h/W_L.csv	**Data Dimension**: 207,173 (R) × 9 (C) **C1**: Latitude; **C2**: Longitude **C3‒C9 [unit: mm]**: 5% quantile for IDF values with the return period of 2, 5, 10, 25, 50, 100, and 500 years. NaN indicates no runoff event caused by a given mechanism.
trend/	Sen’s slope of Mann-Kendall trend in annual maximum series associated with different hydrometeorological mechanisms (W, P, R, ROS, and M) over water years 1951‒2013	
	[duration]/ [mechanism].csv e.g., 24 h/W.csv	**Data Dimension**: 207,173 (R) × 3 (C) **C1**: Latitude; **C2**: Longitude **C3** [unit: mm/year]: Sen’s slope. The value is zero if the trend is not statistically significant.
Driver/	Dominant mechanism of extreme W events with different durations and return periods	
	[duration]/ [return period].csv e.g., 24 h/50 y.csv	**Data Dimension**: 207,173 (R) × 3 (C) **C1**: Latitude; **C2**: Longitude **C3**: Dominant driver IDs; 1 = rain, 2 = ROS, 3 = melt
risk/	Design risk associated with PREC-IDF estimated 100-year extreme events	
	[duration]_ 100 y.csv e.g., 24 h_100y.csv	**Data Dimension**: 207,173 (R) × 3 (C) **C1**: Latitude; **C2**: Longitude **C3** [unit: %]: Bias in 100-year events based on PREC-IDF vs. NG-IDF
SI/	Seasonality of annual maximum W with different durations over water years 1951‒2013	
	[duration]/ W.csv e.g., 24h/W.csv	**Data Dimension**: 207,173 (R) × 4 (C) **C1**: Latitude; **C2**: Longitude; **C3**: Mean date ( = 1 if Oct 1) **C4**: Seasonality index ranging from 0–1

*Note: In “Data File Description”, C = column, R = Row. C[i] indicates the ith column of a data file.

## Technical Validation

The accuracy of estimated *W* and all related datasets depends primarily on the accuracy of daily SWE simulations given that *P* was observational (see Eq. 1). As there exists no data for direct evaluation of NG-IDF curves, we validated SWE simulations against daily SWE observations from 246 SNOTEL stations. Three model performance metrics that compare the simulated and observed SWE were applied: (1) NSE, (2) PEAK.ERR, and (3) PDATE.ERR, which measured the overall goodness-of-fit, peak SWE, and the timing of peak SWE, respectively. Model evaluations (Fig. 2) showed that NSE of daily SWE was greater than 0.6 at 75% of all stations, PEAK.ERR was within ± 25% at 67% of the stations, and PDATE.ERR was within two weeks at 67% of the stations. Overall, the simulations were able to reproduce the observed SWE dynamics at most stations using the cluster-based snow parameters.

## Usage Notes

The NG-IDF datasets listed in Table 3, with no additional data analysis, can be used for a wide variety of applications over spatial scales ranging from local, regional to the CONUS scales. Overall, the presented estimates of extreme events and their characteristics based on long-term observational and simulation records are crucial for understanding flood potential, particularly for cold regions where infrastructure design risk exists from using PREC-IDF curves or NOAA Atlas 14.

**Fig. 3:**
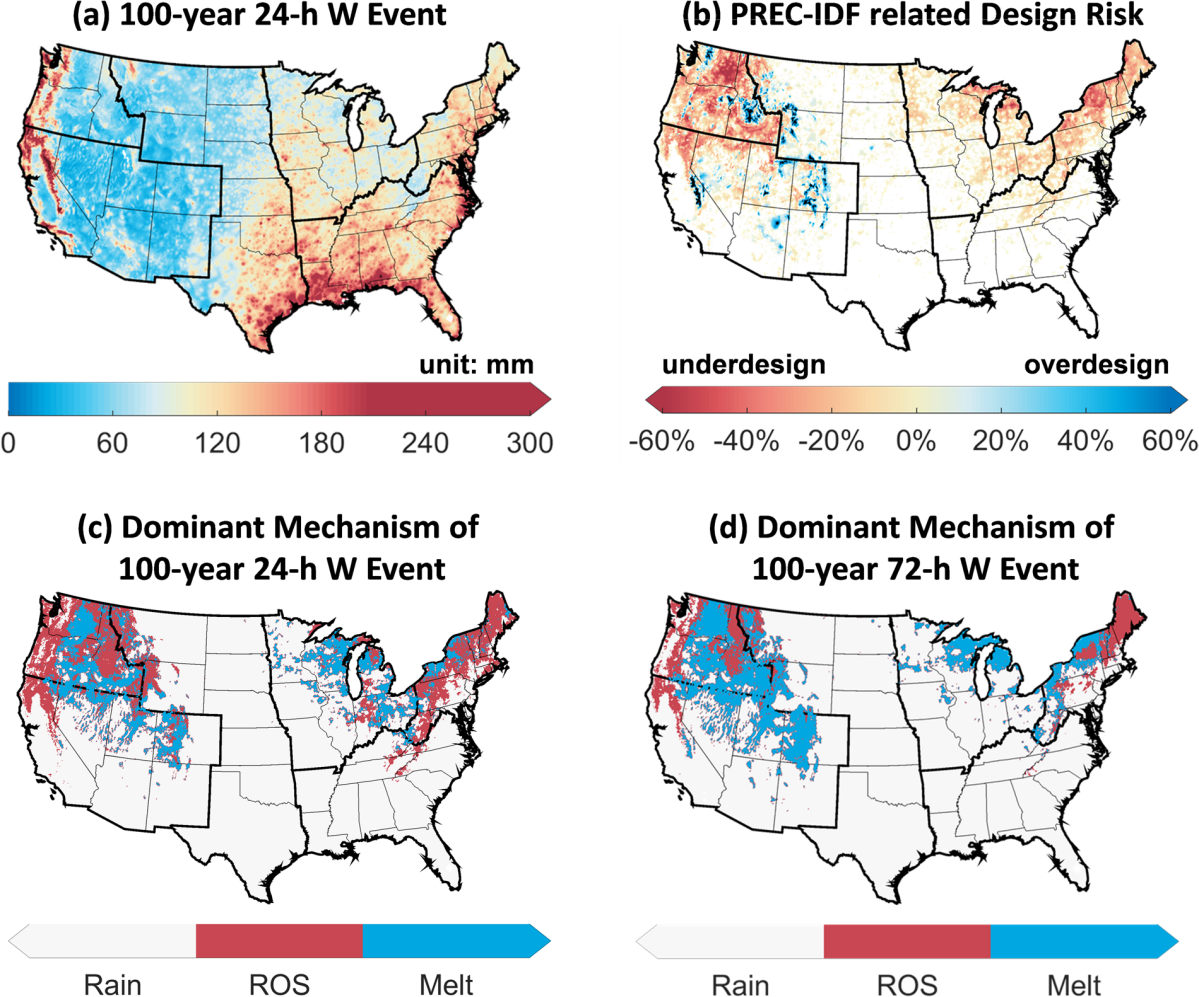
Characteristics of extreme events over the CONUS based on the NG-IDF technique. (**a**) 100-year 24-h extreme events (unit: mm); (**b**) design risk associated with PREC-IDF indicated by the biases of PREC-IDF estimated 100-year 24-h event relative to NG-IDF (underdesign if <−25% and overdesign if >25%); (**c**) dominant mechanism (rain, ROS, or melt) of 24-h extreme events; (**d**) dominant mechanism of 72-h extreme events.

For hydrologic engineering designs and analyses, one can obtain for any location(s) of interest the magnitude of extreme *W* events (Fig. 3a) and their dominant mechanism (Fig. 3c). Through comparing the NG-IDF and PREC-IDF values of extreme events for the same locations, one can determine the magnitude of bias or design risk related to the use of PREC-IDF (Fig. 3b). Information related to the flood seasonality is also key for understanding the generating mechanisms of floods and supporting future flood management. For instance, one can obtain the seasonality index (SI) and the mean timing of AMW events for any location of interest. For locations with a stronger seasonality (i.e., a higher SI), there is less inter-annual variability in the timing of AMW occurrence, and thus the mean date represents better the occurrence dates of AMW from year to year. At broader spatial scales, the datasets can be used for prioritizing locations for flood management and adaptation. As shown in Fig. 3, there is substantial spatial variability in the magnitude of 100-year 24-hour *W* events, which are typically higher in the ROS-dominated Pacific Northwest mountain ranges, and rain-dominated Gulf coastal plains. The dominant mechanism exhibits greater heterogeneity in topographically complex mountainous regions, and there is a shift in the dominant mechanism from ROS to rain or melt for events with a longer duration (72-hour versus 24-hour) (Fig. 3c,d). The presented datasets can also be used for estimating catchment-scale flood responses by coupling the NG-IDF curves with a rainfall-runoff model as demonstrated in prior work^16^.

Lastly, it should be noted that the datasets are subject to a few limitations:

1. Diurnal variability is not represented in the precipitation data used here to force the snow model and construct IDF curves. As a result, our estimates of extreme events are limited to daily or longer durations. While the daily temporal resolution is mostly sufficient to capture snow-related extreme events, short-duration IDF curves based on high-resolution rainfall data are more appropriate for capturing short-duration extremes (e.g., flash floods) or floods in small catchments with fast response times (<24 hours).
2. Although generally smaller compared to sample data uncertainty^8^, the choice of probability distribution can contribute to uncertainties in estimates of extremes^42^. Depending on the usage, one can apply the distribution of choice or ensemble distributions for analysis of extreme *W* events using provided AMW datasets.
3. Greater uncertainties in NG-IDF estimates are expected for locations with lower snow model skill, such as the maritime Pacific Northwest and locations exposed to high winds during winter.
4. Stationarity assumption. Based on the Mann-Kendall test, about 10% of the CONUS shows a statistically significant trend in AMW with any duration of 24, 48, or 72 hours. Hence, the IDF curve estimates based on the stationary assumption are valid for about 90% of the CONUS. For the remaining locations showing a significant trend in AMW, we recommend applying a nonstationary approach for constructing NG-IDF curves. Among a variety of nonstationary approaches^17,43,44^, here we demonstrate the application of the Non-stationary Extreme Value Analysis (NEVA) Software^45^ to construct the NG-IDF curve based on the 24-hour AMW at a location in the Oregon State, where AMW has the highest positive trend of 1.12 mm/decade over the CONUS (Supplementary Fig. 1). In this particular case, the analysis suggests that the stationary assumption may lead to the underestimation of extreme events into the future. With the provided AMW series, one can develop nonstationary IDF curves using the approach of choice.
5. Consideration of land cover. The NG-IDF datasets provided in this study are developed for open conditions (as opposed to forest conditions). Given strong forest-snow interactions^33^ and their implications for streamflows^30^, incorporating different land cover types into the development of NG-IDF datasets is an undergoing research effort. The datasets presented here are used as the baseline for understanding the vegetation impacts or land use change (e.g., urbanization) impacts on NG-IDF curves.

## Supplementary information

Supplemental Information

## Data Availability

The source code of the DHSVM model used for snow simulations can be freely downloaded at https://github.com/pnnl/DHSVM-PNNL. The R programming language was used for developing IDF curves, detecting trend and determining seasonality of annual maximum series, using the following packages: trend^46^, lmom^47^, circular^48^. Source codes that were used to develop and analyze the data are publicly available at https://github.com/Lizzy0Sun/NG-IDF-analysis-code/.

## References

[1] Berghuijs WR, Woods RA, Hutton CJ, Sivapalan M (2016). Dominant flood generating mechanisms across the United States. Geophys. Res. Lett..

[2] Leung LR, Qian Y (2009). Atmospheric rivers induced heavy precipitation and flooding in the western U.S. simulated by the WRF regional climate model. Geophys. Res. Lett..

[3] Li D, Lettenmaier DP, Margulis SA, Andreadis K (2019). The role of rain‐on‐snow in flooding over the conterminous United States. Water Resour. Res..

[4] McCabe GJ, Clark MP, Hay LE (2007). Rain-on-Snow Events in the Western United States. Bull. Am. Meteorol. Soc..

[5] Musselman, K. N., Clark, M. P., Liu, C., Ikeda, K. & Rasmussen, R. Slower snowmelt in a warmer world. **7**, 214–220 (2017).

[6] Ralph FM (2003). The Impact of a Prominent Rain Shadow on Flooding in California’s Santa Cruz Mountains: A CALJET Case Study and Sensitivity to the ENSO Cycle. J. Hydrometeorol..

[7] Perica, S. *et al*. *Precipitation-Frequency Atlas of the United States, NOAA Atlas 14*. (vol. 8, version 2.0, U.S. Dep. of Commer., National Oceanic and Atmospheric Administration, National Weather Service, Silver Spring, Md. (2013).

[8] Yan H (2018). Next-Generation Intensity-Duration-Frequency Curves for Hydrologic Design in Snow-Dominated Environments. Water Resour. Res..

[9] Yan H (2019). Next-Generation Intensity–Duration–Frequency Curves to Reduce Errors in Peak Flood Design. J. Hydrol. Eng..

[10] NCEI. Billiondollar weather and climate disasters. Available at https://www.ncdc.noaa.gov/billions/events/US/1980-2018 (2018).

[11] Hagos SM, Leung LR, Yoon J, Lu J, Gao Y (2016). A projection of changes in landfalling atmospheric river frequency and extreme precipitation over western North America from the Large Ensemble CESM simulations. Geophys. Res. Lett..

[12] Warner MD, Mass CF, Salathé EP (2015). Changes in Winter Atmospheric Rivers along the North American West Coast in CMIP5 Climate Models. J. Hydrometeorol..

[13] Cao Q (2020). Floods due to Atmospheric Rivers along the U.S. West Coast: The Role of Antecedent Soil Moisture in a Warming Climate. J. Hydrometeorol..

[14] Gershunov A (2019). Precipitation regime change in Western North America: The role of Atmospheric Rivers. Sci. Rep..

[15] Hamlet AF (2018). New Observed Data Sets for the Validation of Hydrology and Land Surface Models in Cold Climates. Water Resour. Res..

[16] Yan H (2020). Evaluating next‐generation intensity–duration–frequency curves for design flood estimates in the snow‐dominated western United States. Hydrol. Process..

[17] Yan H, Sun N, Chen X, Wigmosta MS (2020). Next-Generation Intensity-Duration-Frequency Curves for Climate-Resilient Infrastructure Design: Advances and Opportunities. Front. Water.

[18] Wigmosta MS, Vail LW, Lettenmaier DP (1994). A distributed hydrology-vegetation model for complex terrain. Water Resour. Res..

[19] Serreze MC, Clark MP, Frei A (2001). Characteristics of large snowfall events in the montane western United States as examined using snowpack telemetry (SNOTEL) data. Water Resour. Res..

[20] Kendall, M. G. *Rank Correlation Methods*. (1975).

[21] Mann HB (1945). Nonparametric Tests Against Trend. Econometrica.

[22] Sen PK (1968). Estimates of the Regression Coefficient Based on Kendall’s Tau. J. Am. Stat. Assoc..

[23] Hosking, J. R. M. & Wallis, J. R. *Regional Frequency Analysis: An Approach Based on L-Moments*. (Cambridge University Press, Cambridge, U. K., 1997).

[24] Livneh B (2013). A long-term hydrologically based dataset of land surface fluxes and states for the conterminous United States: Update and extensions. J. Clim..

[25] Bohn TJ (2013). Global evaluation of MTCLIM and related algorithms for forcing of ecological and hydrological models. Agric. For. Meteorol..

[26] Serreze MC, Clark MP, Armstrong RL, McGinnis D, Pulwarty RS (1999). Characteristics of the western United States snowpack from snowpack telemetry(SNOTEL) data. Water Resour. Res..

[27] *BCQC SNOTEL data* https://www.pnnl.gov/data-products (2019).

[28] Storck P, Bowling L, Wetherbee P, Lettenmaier D (1998). Application of a GIS-based distributed hydrology model for prediction of forest harvest effects on peak stream flow in the Pacific Northwest. Hydrol. Process..

[29] Sun N (2019). Regional Snow Parameters Estimation for Large‐Domain Hydrological Applications in the Western United States. J. Geophys. Res. Atmos..

[30] Sun N (2018). Evaluating the functionality and streamflow impacts of explicitly modelling forest-snow interactions and canopy gaps in a distributed hydrologic model. Hydrol. Process..

[31] Luce CH, Lopez-Burgos V, Holden Z (2014). Sensitivity of snowpack storage to precipitation and temperature using spatial and temporal analog models. Water Resour. Res..

[32] Lute AC, Luce CH (2017). Are model transferability and complexity antithetical? Insights from validation of a variable-complexity empirical snow model in space and time. Water Resour. Res..

[33] Sun N (2022). Forest Canopy Density Effects on Snowpack across the Climate Gradients of the Western United States Mountain Ranges. Water Resour. Res..

[34] Likas A, Vlassis N, Verbeek JJ (2003). The global k-means clustering algorithm. Pattern Recognit..

[35] Laramie, R. L. & Schaake, J. C. J. *Simulation of the continuous snowmelt process*. (1972).

[36] Freudiger D, Kohn I, Stahl K, Weiler M (2014). Large-scale analysis of changing frequencies of rain-on-snow events with flood-generation potential. Hydrol. Earth Syst. Sci..

[37] Li D, Lettenmaier DP, Margulis SA, Andreadis K (2019). The Role of Rain-on-Snow in Flooding Over the Conterminous United States. Water Resour. Res..

[38] Musselman KN (2018). Projected increases and shifts in rain-on-snow flood risk over western North America. Nat. Clim. Chang..

[39] Burn DH (1997). Catchment similarity for regional flood frequency analysis using seasonality measures. J. Hydrol..

[40] Villarini G (2016). On the seasonality of flooding across the continental United States. Adv. Water Resour..

[41] Sun N (2022). Zenodo.

[42] Yan H, Moradkhani H (2016). Toward more robust extreme flood prediction by Bayesian hierarchical and multimodeling. Nat. Hazards.

[43] Ragno E (2018). Quantifying Changes in Future Intensity‐Duration‐Frequency Curves Using Multimodel Ensemble Simulations. Water Resour. Res..

[44] Hou Z (2019). Incorporating Climate Non-stationarity and Snowmelt Processes in Intensity-Duration-Frequency Analyses with Case Studies in Mountainous Areas. J. Hydrometeorol..

[45] Cheng L, AghaKouchak A (2015). Nonstationary Precipitation Intensity-Duration-Frequency Curves for Infrastructure Design in a Changing Climate. Sci. Rep..

[46] Pohlert, T. Package ‘trend’. (2016).

[47] Hosking, J. R. M. Package ‘lmom’. (2015).

[48] Agostinelli, C. Package ‘circular’. (2017).

