# Supplementary material for: Datasets for characterizing extreme events relevant to hydrologic design over the conterminous United States
Source: Sci Data. 2022 Apr 5;9:154. doi: 10.1038/s41597-022-01221-9 (PMC8983646; doi:10.1038/s41597-022-01221-9)
Supplement: Supplementary file 1 — Supplemental Information [file 41597_2022_1221_MOESM1_ESM.docx]

**Datasets for characterizing extreme events relevant to hydrologic design**

**over the conterminous United States**

Ning Sun, Hongxiang Yan, Mark S. Wigmosta, Andre M. Coleman, L. Ruby Leung, Zhangshuan Hou

**Supplementary Figure**

*
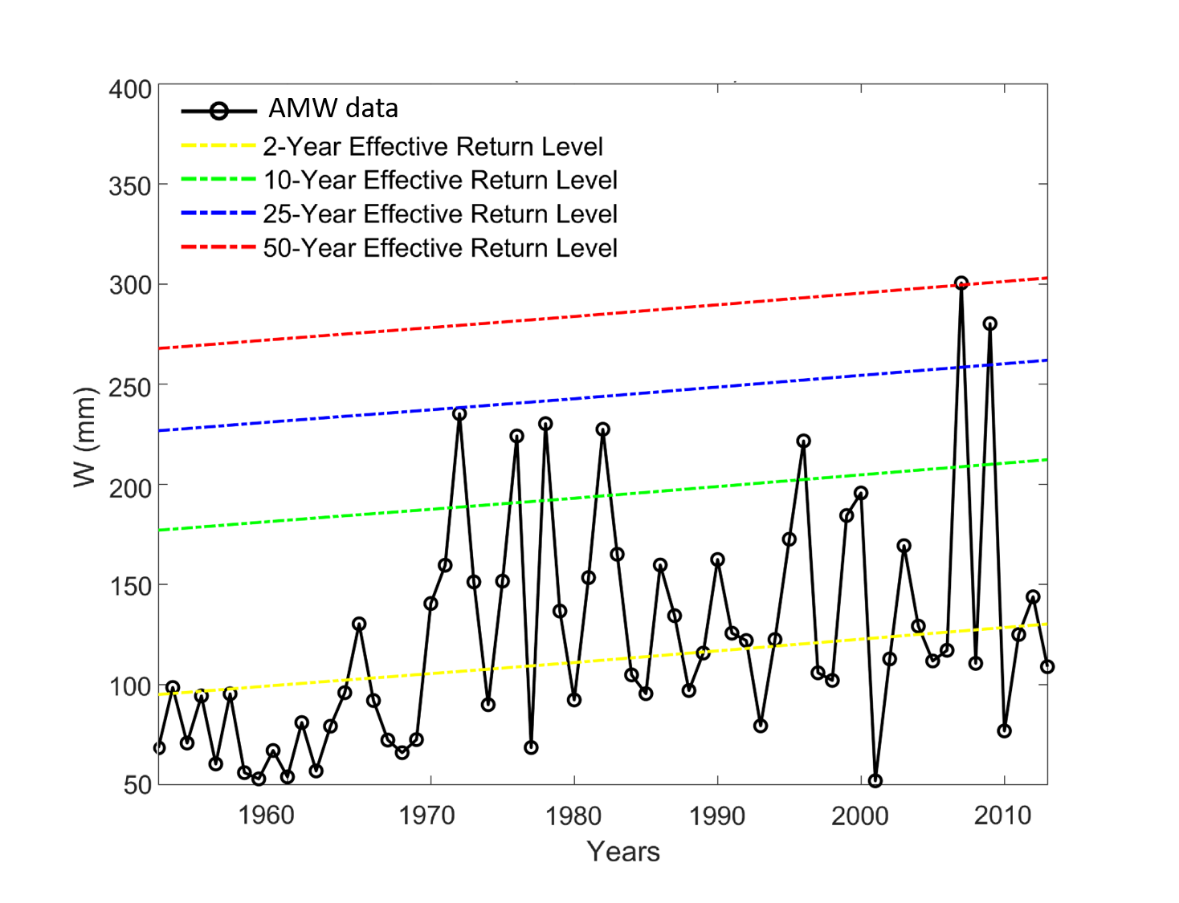
*

Supplementary Figure 1: Estimates of extreme 24-hour W events based on the NG-IDF curve developed using the Non-stationary Extreme Value Analysis (NEVA) approach for a location with the highest positive trend in AMW over the CONUS.
